# Enhanced Reactant Distribution in Redox Flow Cells

**DOI:** 10.3390/molecules24213877

**Published:** 2019-10-28

**Authors:** Nicholas Gurieff, Declan Finn Keogh, Victoria Timchenko, Chris Menictas

**Affiliations:** School of Mechanical and Manufacturing Engineering, UNSW, Sydney, NSW 2052, Australia; n.gurieff@unsw.edu.au (N.G.); d.keogh@unsw.edu.au (D.F.K.); v.timchenko@unsw.edu.au (V.T.)

**Keywords:** vanadium redox flow battery, power density, limiting current, cell geometry, mass transfer, electrolyte mixing, static mixer

## Abstract

Redox flow batteries (RFBs), provide a safe and cost-effective means of storing energy at grid-scale, and will play an important role in the decarbonization of global electricity networks. Several approaches have been explored to improve their efficiency and power density, and recently, cell geometry modification has shown promise in efforts to address mass transport limitations which affect electrochemical and overall system performance. Flow-by electrode configurations have demonstrated significant power density improvements in laboratory testing, however, flow-through designs with conductive felt remain the standard at commercial scale. Concentration gradients exist within these cells, limiting their performance. A new concept of redistributing reactants within the flow frame is introduced in this paper. This research shows a 60% improvement in minimum V^3+^ concentration within simulated vanadium redox flow battery (VRB/VRFB) cells through the application of static mixers. The enhanced reactant distribution showed a cell voltage improvement by reducing concentration overpotential, suggesting a pathway forward to increase limiting current density and cycle efficiencies in RFBs.

## 1. Introduction

The large-scale adoption of renewable energy around the world, required for the decarbonization of power generation, will demand grid-scale energy storage, increasingly in the form of batteries [1]. Batteries are particularly suited to managing distributed, dynamic supply and demand challenges, including variable generators and an increasing number of electric vehicles. Redox flow batteries (RFBs) offer independent power and capacity scaling, long lifetimes, and inherent safety advantages over lithium-ion systems. There is a range of chemistries under development, with an increased focus on organic electrolytes [2], however, all-vanadium redox flow batteries (VRFBs) are already being commercially exploited.

There are exceptions in the form of hybrid systems, however true redox flow batteries decouple power (kW/MW) from the capacity (kWh/MWh) by converting energy in cell stacks and storing energy in liquid electrolyte tanks. The active element or compound, vanadium in the case of VRFBs, reacts while flowing through porous conductive carbon materials within two half-cells separated by a micro-porous separator or ion-selective membrane. Adequate local availability of reactants is essential for reliable and efficient charge and discharge processes, particularly at high and low states of charge (SOC). Material degradation can occur if the system is not managed properly, so flow rates are regulated, and power density is limited [3,4]. Power is a product of cell voltage and current density, making these important factors in RFB system costs [5] and operational efficiencies. 

The cell voltage discussed here, $V_{cell}$, can be described in the form of Equation (1) [3] below:

(1) $$V_{cell}=E^{C}+E^{A}-\eta_{A}-\eta_{C}-iR_{cell}$$

where $\eta_{C}$ is the concentration overpotential, which can dominate when the concentration of a reactant in solution, $C_{b}$, is low. In this case, the limiting current density, $i_{L}$, can be expressed as shown in Equation (2) [4]:

(2) $$i_{L}=nFk_{m}C_{b}$$

Here, the local mass transfer coefficient, $k_{m}$, is a function of velocity, v, as shown in Equation (3) [4] below:

(3) $$k_{m}=1.6\times 10^{-4}\cdot v^{0.4}$$

Despite extensive research and development work, the significance of electrolyte flow and the associated mass-transfer effects has not yet been adequately addressed [6]. Considerable attention has been devoted to material development, electrodes, and reaction mechanisms [7]. Where discussed, the primary focus of cell design has been on the use of flow fields, such as serpentine channels [8], however, critical challenges remain when these concepts are scaled to industrially relevant stack sizes [9]. Flow-through cells remain the standard for this reason, and large-area cells (some greater than 2.5 m^2^) are under development to deliver economies of scale [10]. 

Experimenting with large cells and assessing their internal state is challenging, making numerical modeling an essential tool for the analysis and optimization of these systems. Coupled fluid and electrochemistry models have been used in flow battery research since Shah and co-workers published their dynamic two-dimensional models [11,12,13]. Researchers have applied and developed these models for a range of purposes. A simplified stationary model was published by You et al. [14], then Ma and co-workers applied this to a three-dimensional format [15]. Other groups have included vanadium crossover and water transport through the membrane [16].

Simulation of innovative cell geometries, including trapezoidal [17], and radial designs [18] has recently shown the promise of design modifications to improve performance in flow-through cells. Studies have also explored reducing the cross-sectional area to provide increased electrode compression in addition to a higher flow rate towards the outlet [19]. Subsequent experimental testing on laboratory-scale cells demonstrated improved energy efficiencies with wedge-shaped cells, and a toroidal stack concept based on this concept has been presented [20]. An issue with flow-through cells that persists even with these new geometries, and others like the circular concept [21], is the problem of concentration gradients across the half-cell cross-section between the collector and the membrane [15]. 

**Figure 1:**
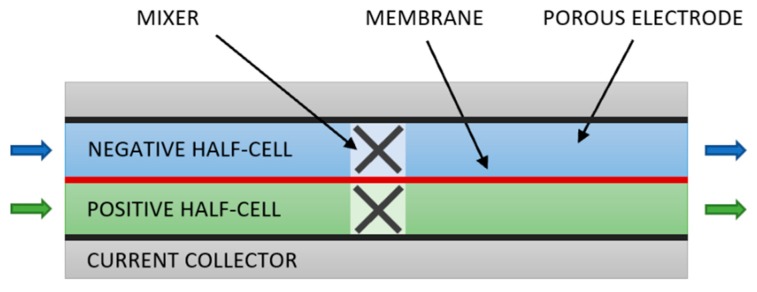
Single-cell diagram showing mixers at halfway point in each half-cell. Note there is a gap in the porous material in the mixer volume.

In the absence of turbulence or engineered architecture, such as the corrugated fluidic networks recently proposed [22], diffusion-limited flow conditions result in reactants being depleted close to boundaries. To address this, we propose applying static mixers, standard equipment in the process industries [23], to flow-through RFB cells using graphite felt electrodes. Mixing is essential in most industrial chemical processes, particularly where velocities are low in laminar flow regimes [24] as in VRFBs. Instead of inserting a mixer in a tube, this would involve adding a mixer (extended in width rather than length) into a gap in the porous electrode material in each half-cell of flow battery as shown in Figure 1.

**Figure 2:**
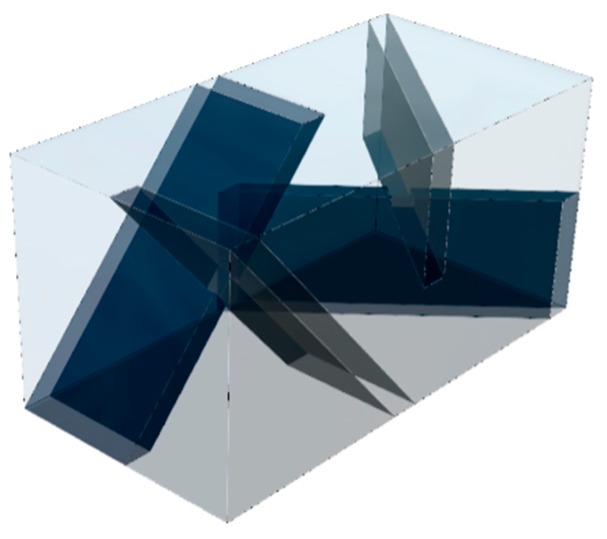
Computer Aided Design (CAD) render of the two-element blade-style mixer used in this study. This configuration has a volume envelope of 2.5 × 2.5 × 5.0 mm.

Static mixers have previously been used in experiments with VRFBs, and were found to reduce mass-transfer limitations, however the work focused on improving performance with slurry electrodes by enabling better charge transfer with particles [25]. Helical mixers, such as the Kenics^TM^ mixer and the conductive mixers used for the slurry electrodes, are commonly found in round pipes and have been studied quite extensively [24,26,27,28]. This style provides flow division and radial mixing however is more challenging to optimize for the thinner rectangular geometry used in efficient stacks. For this reason, a blade-style, low-pressure drop (LPD) type mixer was generated as a starting point for this study. An inline mixer is generally composed of a series of baffle elements, an approach we have applied in this work. A two-element design is shown in Figure 2.

This geometry and others were simulated in a thin (2.5 mm unit-width) section of a flow cell, with symmetry applied to the side boundaries, using validated models with published parameters. In contrast to the mixers used with slurry electrodes [25], the mixers in this research are non-conductive to promote enhanced reactant distribution in the porous media. Results show improved performance when compared to conventional geometry without a mixer during charging at high current density.

## 2. Results

Initial tests were conducted with unit-width negative half-cells by replacing 10% of the porous electrode volume with a mixer. The blade elements of the mixer were then duplicated and rotated to generate geometries of two- and three-element mixers in addition to the single element case. The multi-element domains were longer as the variables were defined in relative terms. 160 mA/cm^2^ current density was applied through the collector boundary to an electrolyte with an inlet concentration of 90% SOC at a flow rate fixed at 5 stoich. Results in comparison to a conventional reference case under the same conditions are shown in Table 1.

Where the improvement in the minimum V^3+^ concentration within the half-cell domain was calculated as shown below in Equation (4):

(4) $$Concentration\;Improvement=\frac{\min\left(c_{V^{3+}}^{mix}\right)-\min\left(c_{V^{3+}}^{ref}\right)}{\min\left(c_{V^{3+}}^{ref}\right)}$$

The improvement in minimum concentration with a third mixer element was insignificant, so the two-element geometry was used in subsequent simulations. The impact of the mixer location was then assessed in 300 mm long half-cells, representative of a 900 cm^2^ cell, where the 5 mm long mixer replaces 1.7% of the electrode volume. Current density and flow rate were defined as before, at 160 mA/cm^2^ and 5 stoich. The results are shown in Table 2. 

**Figure 3:**
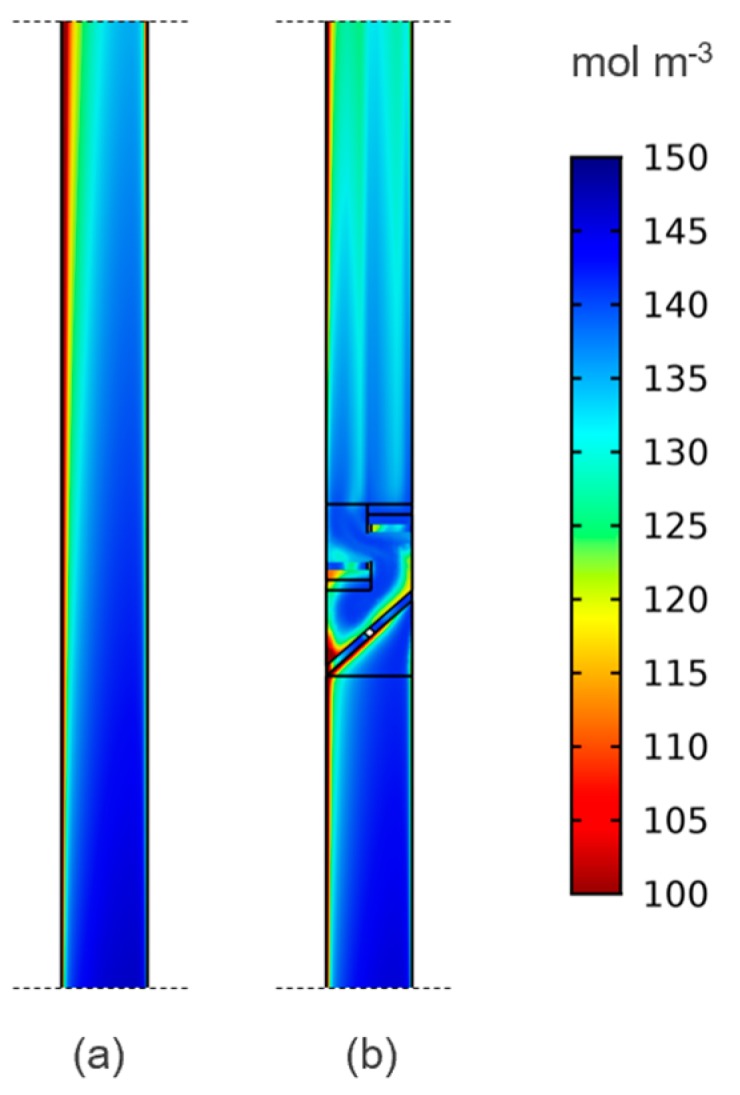
Concentration (mol m^−3^) of V^3+^ on cross-section plane at one-third depth (halfway across a mixer blade) for reference (**a**) and two-element mixer (**b**) cases showing enhanced reactant distribution.

The mid-length position clearly offers the highest improvement when compared to the reference case. The mechanism for the improvement with multiple elements is seen in Figure 3, where the mixer is clearly shown to re-distribute reactants inside the half-cell.

**Figure 4:**
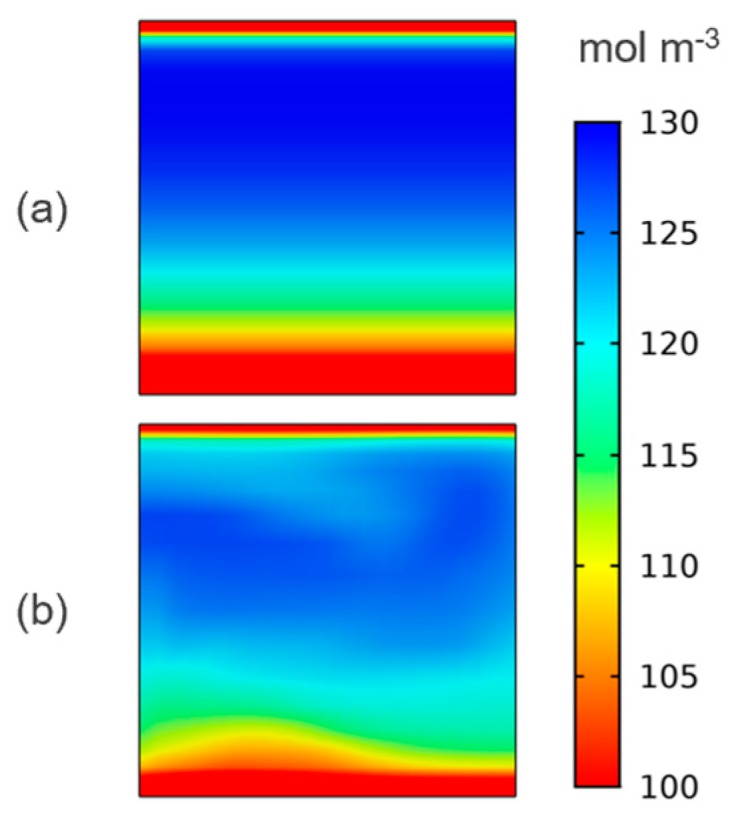
Concentration (mol m^−3^) of V^3+^ at the outlet boundary for reference (**a**) and two-element mixer (**b**) cases showing improved electrolyte utilization during charging at high current density.

This translates through to the outlet boundary, as shown in Figure 4, where the boundary layers are disrupted, and the bulk of the electrolyte is better utilized. This means a higher limiting current density for the same applied conditions, as this is directly proportional to the reactant concentration.

## 3. Discussion

Simulations for a unit-width slice of a 900 cm^2^ single cell showed a 1% improvement in cell voltage when compared to the reference geometry without a mixer. This is small, but a similar magnitude of improvement was seen with wedge-shaped cells applying reducing electrode compression [19], resulting in a 15% increase in energy efficiency during cycling experiments [20]. It is also worth noting that this was achieved while removing 1.7% of the porous electrode, rather than increasing current collector surface area with conductive mixers as applied to slurry electrodes [25]. The conductive helical mixers used in that study also delivered improved mass transport, however, the energy efficiency of thicker slurry cells remains lower than the thinner contemporary commercial stacks with carbon felt, which is the proposed application for the mixers in this study.

Table 3 shows that the pressure drop relative to a traditional geometry is higher at increased flow rates, which is expected, a mixer does have an energy cost. It is noted, however, that based on the improved reactant distribution, the flow rate could be reduced for the same current and the mixer geometry can also be optimized to improve mixing while reducing flow resistance. This could also be countered by applying varied compression over the length of the cell, which has been shown to reduce pressure drop and improve electrochemical performance [19,20].

**Figure 5:**
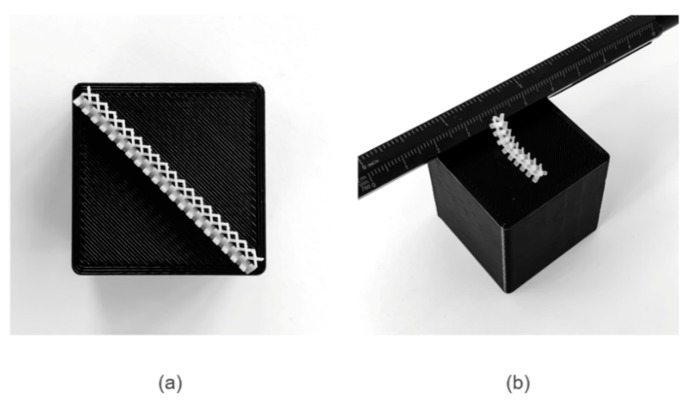
Prototype two-element mixers additively manufactured using (**a**) stereolithography (SLA) in rigid form and (**b**) material jetting (MJP) with flexible material.

Membrane punctures could conceivably be an issue with combinations of a hard mixer and thin membranes, as even carbon fibers can protrude [29]. The risk from the mixer itself, however, is mitigated by the surrounding compressed porous material, which would prevent excessive contact pressure between the mixer and the membrane. The separator could also be reinforced, particularly in larger cells, and the mixer could be produced from a soft material. Prototype two-element mixers additively manufactured in rigid Accura^®^ Xtreme™ and flexible VisiJet^®^ CE-NT material are shown below in Figure 5. The rigid stereolithography (SLA) material has a shore hardness of 86D [30] while the flexible material jetting (MJP) polymer is 27-33A [31]. Selecting appropriate membrane and mixer materials for an industrial flow battery application is complex and will depend on a range of factors, including commercial decisions on cost and durability, as discussed at the International Flow Battery Forum [32].

**Figure 6:**
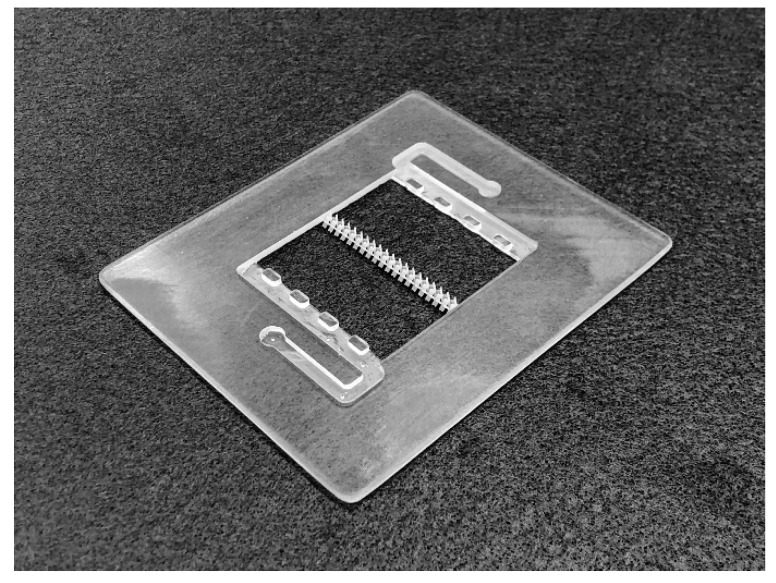
Prototype mixer in a 25 cm^2^ flow frame. Both parts were additively manufactured using stereolithography (SLA), in rigid grey and transparent material, respectively.

Electrode intrusion could also affect the performance, however, the impact is likely to be positive due to the increased reaction volume [33]. Further research will be required to assess the impact of this on mixing performance and will seek to demonstrate this concept experimentally in laboratory-scale cells, and Figure 6 shows preliminary work to this end with the prototype two-element mixer positioned in a flow frame manufactured with the same 3D printing process. A longer cell is planned to better examine the effects expected in large stacks, where gradients are more pronounced.

Future work will also explore the combination of this innovation with wedge-shaped cells–the two innovations are not mutually exclusive, and as discussed above the combination of both promises added benefits for redox flow batteries. Investigation of other geometries and conductive materials, like those used with slurry electrodes [25], is also to be conducted. Simulation provides an accessible means to explore these options and complement laboratory cycling experiments, which can also assess the impact of this innovation on flow cell durability. 

In summary, more research is required to assess and optimize all the coupled variables, however the results presented here suggest this would be worthwhile. The addition of a mixer in a redox flow battery cell has potential to improve the electrochemical performance, particularly in the larger cells that are used in commercial systems.

## 4. Materials and Methods 

The methodology for this work was based on techniques applied in previously published research [18,19]. A concise overview and key parameters are provided here, further details are available in the references.

Governing equations were applied based on the approach developed by Shah et al. [11] and You et al. [14]. Ion flux is described by the Nernst-Planck equations and the Butler-Volmer law is used to define electrode reaction kinetics, while Darcy’s law was applied to give the velocity in the porous electrode. Some elements of the model were taken from Knehr et al. [16] in relation to fluid parameters and the treatment of the boundaries between the membrane and electrodes. Selected SOC values were simulated for model validation, as described by You et al. and Zheng et al. [34].

**Figure 7:**
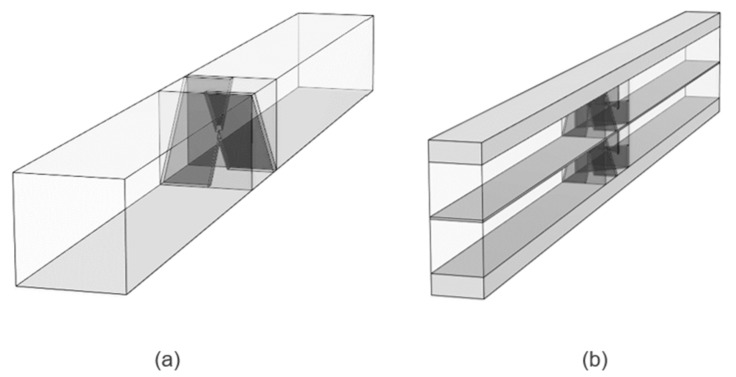
CAD renders of (**a**) unit-width half-cell with a single element mixer and (**b**) a shortened unit-width full-cell with two-element blade-style mixers in both half-cells.

Simplified half-cell models (one geometry shown in Figure 7a) were used for the initial studies to assess the impact of varying flow rate, the number of mixer elements, and the location of the mixer in the cell. Full-cell models (geometry with mixers, truncated for easy viewing, shown in Figure 7b) were used to assess the effect of the mixer on cell voltage. Symmetry boundaries were used on both sides of the unit-width geometry to minimize the computational domain.

Mapped meshes with elements biased towards the outlet and current collector boundaries were used for the conventional rectangular geometry without mixers. The volume surrounding the mixers was discretized with unstructured meshes. Mesh refinement studies were conducted to determine the required resolution to achieve convergence for minimum concentration values, which are dependent on both convective mass transfer and electrochemical processes. Numbers of elements in the order of 200,000 were found to provide acceptable resolution. 

The coupled electrochemical and fluid models and were solved using the finite element method with software package COMSOL Multiphysics with the UNSW Sydney computational cluster, Katana. A combination of flow and current distribution interfaces were used to implement the convection-diffusion, general-form, and ordinary differential equations. Except where stated elsewhere, the general parameters shown in Table 4 were applied. 

Inlet velocity was defined as a multiple of the stoichiometric requirement based on the applied current, which is a function of cell size and current density. Key parameters for positive and negative fluids are shown below in Table 5. The electrolyte is assumed to have constant physical properties.

Electrode and current collector properties are shown below in Table 6. The Carmen–Kozeny equation was used to define the permeability of the electrodes as a function of porosity.

Electrochemical properties used are summarised in Table 7 for reference. A Bruggeman correction was applied to diffusion coefficients and electrical conductivity parameters.

The full-cell model implemented here with parameters defined by You et al. [14], including a 140 mV voltage correction for considerations not included in the simplified model such as contact resistances, showed good agreement with the experimental data published in their work. The values did not vary significantly from those previously reported [19], with an average error of 1% and a maximum error of 3%. A comparison between the full-cell and half-cell models showed no significant difference in key values (maximum 0.2% variation for minimum V^3+^ concentration).

Performance parameters were obtained through post-processing in COMSOL. Derived values were introduced to provide the minimum V^3+^ concentration over the negative electrode cross-section. A boundary probe was used to obtain the electric potential on the current collector boundary on the positive half-cell. This boundary is defined with an electrode current boundary condition set at the applied average current density, while the negative is defined as electric ground. Differential pressure was obtained by subtracting the average absolute pressure at the inlet from the absolute pressure at the outlet boundary.

## Figures and Tables

**Table 1:** Improvement in minimum V^3+^ concentration with one-, two-, and three-element mixers relative to conventional geometry.

Geometry	Concentration Improvement
1 Element	7%
2 Element	41%
3 Element	42%

**Table 2:** Improvement in minimum V^3+^ concentration with two-element mixers at three different locations relative to conventional geometry.

Location	Concentration Improvement
1/3 Position	36%
1/2 Position	60%
2/3 Position	44%

**Table 3:** Change in pressure drop across a 300 mm long unit-width half-cell relative to conventional geometry over a range of flow rates defined as a multiple of the stoichiometric requirement (stoich).

Flow Rate	Pressure Drop
1	1%
5	−2%
10	−4%

**Table 4:** General operating parameters.

Parameter	Symbol	Value	Unit
Outlet pressure	P	0	Pa
Temperature	T	293.15	K
Current density	i	160	mA cm^−2^
State of Charge	SOC	90	-
Half-cell electrode thickness	h	2.5 × 10^−3^	m
Domain width	w	2.5 × 10^−3^	m
Current collector thickness	h	1.0 × 10^−3^	m
Membrane thickness	d	0.1 × 10^−3^	m

**Table 5:** Fluid model parameters from Knehr et al. [16].

Parameter	Symbol	Value	Unit
Dynamic viscosity (negative electrolyte)	$\mu_{-}$	0.0025	Pa s
Dynamic viscosity (positive electrolyte)	$\mu_{+}$	0.005	Pa s
Density (negative electrolyte)	$\rho_{-}$	1300	kg m^−3^
Density (positive electrolyte)	$\rho_{+}$	1350	kg m^−3^

**Table 6:** Electrode and current collector parameters from Knehr et al. [16].

Parameter	Symbol	Value	Unit
Conductivity of current collector	$\sigma_{s}^{cc}$	1000	S m^−1^
Conductivity of electrode	$\sigma_{s}^{e}$	66.7	S m^−1^
Porosity	ϵ	0.929	-
Mean pore radius	$r_{p}$	50.3 × 10^−6^	m
Kozeny–Carman constant	$k_{CK}$	180	-

**Table 7:** Electrochemical parameters from You et al. [14].

Parameter	Symbol	Value	Unit
V^2+^ diffusion coefficient	$D_{V^{2+}}$	2.4 × 10^−10^	m^2^ s^−1^
V^3+^ diffusion coefficient	$D_{V^{3+}}$	2.4 × 10^−10^	m^2^ s^−1^
VO^2+^ diffusion coefficient	$D_{VO^{2+}\;}$	3.9 × 10^−10^	m^2^ s^−1^
VO_2_^+^ diffusion coefficient	$D_{VO_{2}^{+}}$	3.9 × 10^−10^	m^2^ s^−1^
Proton diffusion coefficient	$D_{H^{+}\;}$	9.312 × 10^−9^	m^2^ s^−1^
Initial vanadium concentration	$c^{0}$	1500	mol m^−3^
Initial proton concentration (negative)	$c_{n_{H+}}^{0}$	4500	mol m^−3^
Initial proton concentration (positive)	$c_{p_{H+}}^{0}$	6000	mol m^−3^
Standard reaction rate constant (negative)	$k_{c}$	1.7 × 10^−7^	m s^−1^
Standard reaction rate constant (positive)	$k_{c}$	6.8 × 10^−7^	m s^−1^
Anodic transfer coefficient	$\alpha_{a}$	0.5	–
Cathodic transfer coefficient	$\alpha_{c}$	0.5	–
Equilibrium potential: V^2+^/V^3+^	$E_{c,-}^{\prime }$	−0.255	V
Equilibrium potential: VO^2+^/VO_2_^+^	$E_{c,+}^{\prime }$	1.004	V
